# Meiofauna improve oxygenation and accelerate sulfide removal in the seasonally hypoxic seabed

**DOI:** 10.1016/j.marenvres.2020.104968

**Published:** 2020-07

**Authors:** Stefano Bonaglia, Johanna Hedberg, Ugo Marzocchi, Sven Iburg, Ronnie N. Glud, Francisco J.A. Nascimento

**Affiliations:** a Department of Ecology, Environment and Plant Sciences, Stockholm University, Stockholm, Sweden; b Nordcee, Department of Biology, University of Southern Denmark, Denmark; c Department of Biosciences, Center for Electromicrobiology, Aarhus University, Aarhus, Denmark; d Department of Ocean and Environmental Sciences, Tokyo University of Marine Science and Technology, Tokyo, Japan

**Keywords:** Hypoxia, Sediment, Meiofauna, Sulfide oxidation, Oxygen penetration, Nematode, Cable bacteria, 16S rRNA sequencing, Microbial communities

## Abstract

Oxygen depleted areas are widespread in the marine realm. Unlike macrofauna, meiofauna are abundant in hypoxic sediments. We studied to what extent meiofauna affect oxygen availability, sulfide removal and microbial communities. Meiofauna were extracted alive and added to intact sediments simulating abundance gradients previously reported in the area. A total of 324 porewater microprofiles were recorded over a 3-week incubation period and microbial community structure and cable bacteria densities were determined at the end of the experiment. At high abundances meiofauna activity deepened oxygen penetration by 85%, 59%, and 62% after 5, 14, and 22 days, respectively, compared to control sediment with scarce meiofauna. After 6 days, meiofauna increased the volume of oxidized, sulfide-free sediment by 68% and reduced sulfide fluxes from 8.8 to 0.4 mmol m^−2^ d^−1^. After 15 days, the difference with the control attenuated due to the presence of a cable bacteria population, which facilitated sulfides oxidation in all treatments. 16S rRNA gene analysis revealed that meiofauna affected microbial community structure (beta diversity). Thus, meiofauna bioturbation plays an important role in deepening oxygen penetration, counteracting euxinia and in structuring microbial diversity of hypoxic sediments. Co-existence with cable bacteria demonstrates neutralism interaction between these two ecosystem engineers.

## Highlights

• High meiofauna abundances increase the volume of oxidized, sulfide-free experimental sediment.
• At high abundances, meiofauna significantly deepen oxygen penetration.
• Meiofauna can coexist with cable bacteria in seasonally hypoxic sediments.
• Microbial community structure is significantly altered by meiofauna.

## 1 Introduction

The seabed is densely inhabited by benthic ecosystem engineers (Lohrer et al., 2004; Solan et al., 2004), which are generally classified based on their body size. Macrofauna are invertebrates larger than 1 mm while meiofauna are between 40 μm and 1 mm (Giere, 2009). High abundances, fast biomass turnover and continuous bioturbation activity by meiofauna profoundly influence sediment geochemical processes such as organic matter mineralization (Nascimento et al., 2012) and denitrification (Bonaglia et al., 2014), thereby exerting vital ecosystem functions (Schratzberger and Ingels, 2018). Oxygen (O_2_) levels are diminishing globally both in open oceans and in coastal waters (Breitburg et al., 2018). Deoxygenation is exacerbated by water stratification, enclosed morphology of the water system, nutrient input from land (eutrophication) and climate change (Breitburg et al., 2018; Diaz and Rosenberg, 2008; Elmgren, 2012). Hypoxic conditions (≤2 ml O_2_ L^−1^) have a direct negative impact on aquatic life (Diaz and Rosenberg, 2008). As a consequence, macrofauna can only be present in low abundances or even absent in hypoxic sediments (Diaz and Rosenberg, 1995; Norkko et al., 2019).

Meiofauna, however, present much stronger adaptation to low oxygen conditions and sulfide presence (Wetzel et al., 2001). Meiobenthic organisms—mainly nematodes—are found at abundances ranging between 9 and 3452 ind. 10^−3^ m^-2^ in hypoxic sediments of the Baltic Sea, Gulf of Mexico and Black Sea (Elmgren, 1975; Sergeeva et al., 2013; Wetzel et al., 2001). As such, meiofauna are often the sole bioturbators present in seasonally hypoxic systems, and their activity might be important in mediating benthic biogeochemical processes. Although they dominate in abundance, biomass and diversity in hypoxic conditions, the focus of the research has so far been on the colonization and bioturbation by larger macrofaunal animals (e.g., Bonaglia et al., 2019; Ekeroth et al., 2016). To our knowledge, no studies have investigated how meiofauna bioturbation affects biogeochemical processes in systems that frequently experience hypoxic conditions.

Seasonally hypoxic marine environments exhibit high concentrations of free sulfides (H_2_S) in the sediment porewater, which may escape to the water column (Brüchert et al., 2003; Jørgensen et al., 2004). Free H_2_S is toxic to macrofauna (Vaquer-Sunyer and Duarte, 2010), as it hinders enzymatic processes related to energy acquisition in the mitochondria (Nicholls, 1975). Marine nematodes, which are the predominant meiofaunal organisms in soft sediments (Heip et al., 1982), are often found in high abundances in sulfidic sediments and in a number of cases nematode species have been found in relation with chemosynthetic symbionts oxidizing sulfide (Bellec et al., 2019; Musat et al., 2007; Ott et al., 1991; Tchesunov et al., 2012). Some nematode genera like *Sabatieria* and *Oncholaimus* can migrate many times per day between the oxic and the sulfidic sediment layers. Other taxa like Stilbonematidae and Astomonematinae use their long, filiform body to cover the distance between micropatches of oxygen and sulfides. It is believed that these nematodes stimulate the activity of their symbionts from which they receive nutrition (Jensen, 1987; Ott et al., 1991). Meiofauna activity has been shown to have an important regulatory effect on bacterial activity and community structure (Giere, 2009) through predation (Montagna, 1984) bioturbation that alters solute exchange (Aller and Aller, 1992) and fast turnover rates that quickly return nutrients to bacteria (Coull, 1999).

Centimeters-long filamentous bacteria, namely cable bacteria (Pfeffer et al., 2012), can also exert a profound impact on the biogeochemistry of sulfide-rich sediments. By electrically coupling sulfide oxidation to oxygen or nitrate reduction over centimeter distance (Marzocchi et al., 2014; Nielsen et al., 2010), cable bacteria effectively void the sediment of free sulfide and can delay its release once anoxic conditions return in the bottom water (Seitaj et al., 2015). These organisms may also generate a characteristic pH peak in the oxic layer due to electrochemical oxygen reduction (Nielsen et al., 2010). Similar to meiofauna, cable bacteria can therefore be important ecosystem engineers, and have been recently reported in hypoxic Baltic Sea sediments devoid of macrofauna (Hermans et al., 2019; Marzocchi et al., 2018). Sediment mixing by burrowing infauna is thought to inhibit cable bacteria community (Malkin et al., 2014). Meiofauna might also negatively impact cable bacteria activity via mechanical disruption of the filaments or via predation. However, the possible interaction between meiofauna and cable bacteria has not yet been explored.

Here, we conducted an experimental study using intact sediment cores from an 80-m-deep coastal Baltic Sea basin affected by seasonal hypoxia and tested whether increasing abundances of meiofauna have an effect on sediment geochemistry (O_2_, pH and H_2_S) and microbial communities, with emphasis on the cable bacteria population. Specifically, we hypothesized that: (1) high induced meiofauna abundance increase O_2_ penetration and lower H_2_S concentrations; (2) meiofauna coexist with cable bacteria; and (3) meiofauna significantly change microbial community structure in hypoxic sediment. We avoided heavy sediment manipulation (i.e., sieving) of the sediment cores—which alters geochemical and redox gradients—but rather extracted meiofauna alive from additional sediment cores. Sediment geochemical properties were assessed weekly over a 3-week incubation period. At the end of the experiment, meiofauna and filaments of cable bacteria were counted, while microbial communities were assessed by 16S rRNA sequencing. For the first time we tested meiofauna effects on biogeochemical processes in seasonally hypoxic sediments and studied interactions between meiofauna and cable bacteria at near in situ conditions.

## 2 Materials and methods

### 2.1 Sampling site and sediment collection

The Baltic Sea is the largest fjord system in the world, receiving waters from over 200 rivers, and it is characterized by a gradient in salinity, which reaches approximately 3 in the north and 15 in the south (Snoeijs-Leijonmalm et al., 2017). Its water masses are densely stratified due to its enclosed nature and scarce inflows of marine waters from the North Sea. Stratification combined with high density population in the catchment result in eutrophication and in temporal or permanent oxygen depletion in the deeper basins, which generally occurs below the permanent halocline situated at 60–80 m depth (Hansson et al., 2011). The portion of the Baltic seafloor below these depths has an area of ~60,000 km^2^ (Carstensen et al., 2014) and is largely or completely devoid of macrobenthos (Diaz and Rosenberg, 1995; Norkko et al., 2019), but still inhabited by resistant meiofauna such as nematodes, ostracods and kinorhynchs (Elmgren, 1975; Modig and Ólafsson, 1998). Interestingly, meiofauna abundances can reach here up to 3452 ind. 10 cm^−2^ (Elmgren, 1975).

Sampling was carried out in October 2018 at a 80-m-deep Baltic Proper basin, Tvären, a crater structure formed by a meteorite impact in the Middle Ordovician (Lindström et al., 1994). Bottom water temperature was 8 °C, salinity 7 and oxygen concentration 280 μM equivalent to 76% O_2_ saturation. Sediment cores for the experimental setup were collected at a 77-m site (58 46.3116 N, 017 25.8471 E) by means of a multicorer. Multicore liners (n = 15; height 50 cm, surface area 63.6 cm^2^) were subsampled with smaller PVC liners (height 30 cm, surface area 16.6 cm^2^). The sediment at this station was soft, black mud smelling sulfide with a 2‒3-mm thick brown layer on the top. Macrofauna were not present in the sediment, due to seasonal hypoxia/anoxia and sulfide presence.

Additional multicores (n = 6) were collected for alive meiofauna extractions at a shallower nearby site (50-m deep; 58 47.0643 N, 017 24.6370 E) that presented the same salinity, temperature and O_2_ as the deeper site. These cores were sliced onboard and the 0–2 cm layer placed in large petri dish with overlying bottom water. Additional sand-filtered bottom water was collected (50 L). Water, intact cores and core slices were kept at in situ temperature while transported to the laboratories of Stockholm University, within 2–3 h upon collection. Intact cores were transferred to an incubation tank filled with 20-L bottom water, equipped with water and air pumps, and placed in a thermo-constant room at 8 °C.

### 2.2 Meiofauna extraction and experimental setup

Meiofauna extractions were carried out using the sieving and density extraction method previously described (Bonaglia et al., 2014; Nascimento et al., 2012). In brief, each multicore slice was passed through a 40-μm sieve. The meiofauna and sediment retained on this sieve were then submerged for 5 min in a 7% solution of MgCl_2_, rinsed with bottom water, and washed into an Erlenmeyer flask containing Levasil® 200A 40% colloidal silica solution (H. C. Starck SilicaSol GmbH) with a density of 1.21. The flask was turned upside down several times and was then left to settle for 5 min. The top 3–4 cm of Levasil® solution was poured onto a 40-μm sieve, the retained meiofauna and sediment were rinsed with bottom water, and washed into a 50 ml Falcon tube. The sieved Levasil® was poured back into the flask with the remaining sediment and meiofauna, and the procedure was then repeated twice, the last repetition with a 20 min settling time. The Levasil® solution left in the flask after extractions was poured through a 250-μm sieve on top of a 165-μm sieve, to remove larger particles, to gather Ostracods and to confirm absence of macrofauna. The sediment and meiofauna retained in the 165-μm sieve, was rinsed with in situ bottom water and washed into a 50 ml Falcon tube. The average extraction efficiency of a similar procedure using similar sediment was 98% for nematodes, 87% for copepods, and 71% for other groups combined (Olafsson and Elmgren, 1991). The extracted meiofauna were stored in the climate chamber at 8 °C.

The extracts from the six sediment slices were divided and added to the intact cores to create a gradient in meiofauna abundances. Temporary hypoxic-anoxic sediments at these depths have meiofauna abundances of 24–3452 ind. 10^−3^ m^-2^ (Elmgren, 1975). Our sediment had meiofauna abundances in the lower end of this range (128 ind. 10^−3^ m^-2^) because Tvären undergoes oxygenation only in September–October after stagnation and bottom water anoxia in the summer (Bonaglia et al., 2017). We thus kept three unmanipulated cores (control) and added meiofauna aliquots—with abundances estimated from the extracts—to the other cores in order to increase meiofauna by 1.5-fold (low meiofauna), 6-fold (medium meiofauna) and 16-fold (high meiofauna) the original abundances. The meiofauna extracts were carefully poured on top of each sediment core and were left to settle before submerging them again into the water of the incubation tank. All treatments had three replicates each.

### 2.3 Microsensor profiling for O_2_, pH and H_2_S

Sediment microprofiles for O_2_, pH and H_2_S were measured in each sediment unit (n = 12) to examine how sediment chemistry was affected by the different abundances of meiofauna. Profiling was performed using 50 or 100-μm tip microsensors (OX-50, pH-100, H2S-100, Unisense, Denmark) that were mounted onto a motorized micromanipulator (MM33, Unisense, Denmark). Signals were recorded on a four-channel multimeter (Unisense, Denmark) communicating with a laptop. Profiles for O_2_ were measured at a vertical resolution of 100 μm, while pH and H_2_S profiles were made using a vertical resolution of 250 μm. A water column of approximately 5 cm above the sediment was circulated by a gentle flow of air towards the water surface with a 45° angle. This allowed to maintain a constant diffusive boundary layer during measurements. Profiles were made once a week for three weeks with three replicate profiles in each core per each solute. A total of 324 O_2_, H_2_S and pH profiles were recorded for this study.

Sensors were calibrated on each day of measurement. The O_2_ sensor was calibrated using a two-point calibration procedure in O_2_ saturated bottom water (100% O_2_) and inside the sediment (0% O_2_). The H_2_S sensor was calibrated in fresh anoxic solutions containing increasing amounts of a 10 mM Na_2_S stock solution. The pH sensor was calibrated using commercial standards of pH 4.01, 7.00 and 10.01.

Oxygen penetration depth (OPD) was defined as the depth where O_2_ concentration was steadily < 1 μM. The H_2_S horizon was defined as the depth where H_2_S was detectable (>1 μM). Total hydrogen sulfide concentrations (ΣH_2_S = [H_2_S] + [HS^−^]) were calculated at each depth from the measured pH and H_2_S values and the known dissociation constant (pK1) of H_2_S/HS^−^ (Jeroschewski et al., 1996). Fluxes (J) of O_2_ and ΣH_2_S between sediment and water were calculated from the concentration profiles in the sediment using Fick's first law:

(1) $$J=-\phi \;\left(D_{\text{s}}+D_{b}\right)\;\frac{\partial C}{\partial x}$$

where ϕ is sediment porosity; D_s_ and D_b_ are molecular diffusivity and biodiffusivity of solutes in the sediment, respectively; ∂C/∂x is the concentration gradient obtained by linear regression of the solute concentrations vs. depth intervals. D_s_ was estimated as a function of porosity:

(2) $$D_{\text{s}}=\phi^{2}\;\times \;D_{\text{mol}}$$

where D_mol_ is the free diffusion solute coefficient at infinite dilution (Ullman and Aller, 1982). The bioturbation coefficient D_b_ is dependent on the actual meiofauna abundance in the sediment and was obtained by plotting four previously published D_b_ values (Bonaglia et al., 2014; Rysgaard et al., 2000) against meiofauna abundances in the experimental sediments. Working D_b_ for each meiofauna treatment was extrapolated from the resulting linear relationship (Fig. S1).

### 2.4 Experiment termination, meiofauna counting and sediment properties

All experimental cores were sliced into two slices (0–1 cm and 1–2 cm). The top slice was gently homogenized and: 2-ml sediment were sampled for 16S rRNA gene sequencing and stored at −20 °C until DNA extraction; 0.5-ml sediment was sampled, mixed with 0.5 ml 99.8% ethanol and stored at −20 °C for later cable bacteria analyses; the remaining sediment portion from the 0–1 cm slice and the entire 1–2 cm slice were preserved in a 5% formaldehyde solution containing rose Bengal, and kept at 8 °C for meiofauna extraction and counting. Extraction was performed as described in section 2.2. Extracted meiofauna was counted using a 60x binocular stereo microscope (Leica M80) and identified to the highest possible taxonomic level. Additional sediment aliquots were dried at 75 °C for 24 h and subsequently treated at 550 °C for 5 h to calculate porosity and organic matter content (loss of ignition), respectively.

### 2.5 Analyses of cable bacteria by fluorescence in situ hybridization

Fluorescence in situ hybridization (FISH) was conducted to estimate the abundance of cable bacteria at the end of the experiment. Samples were homogenized by mild ultrasonic treatment of ~30% power with 3 cycles x 20 s with 10 s between cycles. Subsamples of 100 μl were added to a mixture of 880 μl of sodium pyrophosphate buffer and 20 μl of agarose 1%. FISH was performed according to previous published protocols (Pernthaler et al., 2001). EUB338 probemixture (Daims et al., 1999) and probe NON338 (Wallner et al., 1993) were used as positive and negative controls, respectively, and probe DSB706 (Loy et al., 2002). Samples were counterstained with the general DNA stain 4’,6-diamidino-2-phenylindole (DAPI). Microscopic analysis was performed on an Axiovert 200 inverted microscope for transmitted light and epifluorescence (Carl Zeiss, Göttingen, Germany) using a 40x lens (with 10x in the binocular equals a total magnification of 400 times). Filament densities of cable bacteria within the sediment (meters of filaments per cm^2^ sediment) were estimated according to the line-intersection method as in Pfeffer et al. (2012).

### 2.6 Microbial analyses by 16S rRNA sequencing and bioinformatics

DNA was extracted from 0.25 g of sediment samples using the DNeasy PowerSoil Kit (QIAGEN) and stored at −20 °C until library preparation. This quantity of sediment is insufficient to reliably capture metazoan eDNA in Baltic soft sediments (Nascimento et al., 2018). As such the eDNA from the ectobiome of meiofauna was deemed to be insignificant for bacterial community structure. The V3–V4 region on the 16S rRNA gene marker for each sample was amplified in triplicate using the 341F (CCTACGGGNGGCWGCAG) and 805R (GACTACHVGGGTATCTAATCC) primers (Herlemann et al., 2011). Polymerase chain reaction (PCR) round one conditions were: 30 s at 98 °C, followed by 12 cycles of 10 s at 98 °C, 30 s at 50 °C, 30 s at 72 °C. The first round amplicons were cleaned with the addition of 0.1 μl Exonuclease 1 (New England BioLabs) and 0.2 μl Thermosensitive Alkaline Phosphatase (Promega), and to finalize the reaction the amplicons were incubated for 15 min at 37 °C, followed by 15 min at 74 °C. PCR round two was performed to dual barcode the amplicons with the use of indexing primers as described by Hu et al. (2016). Conditions during this second thermocycling round were: 3 min at 95 °C, 15 cycles of 30 s at 95 °C, 30 s at 55 °C, 30 s at 72 °C, and 5 min at 72 °C. Triplicates from each sample were then visualized by gel electrophoresis, pooled, purified with Agencourt AMPure XP magnetic beads (Beckman Coulter) and quantified with Qubit (Invitrogen). The purified amplicons were then pooled in equimolar quantities and sequenced in both directions on an Illumina MiSeq platform at the National Genomics Institute (NGI-Stockholm, Sweden). The raw sequence data were uploaded to NCBI GenBank and are available at the BioProject number PRJNA595085.

16S rRNA sequence reads were demultiplexed by NGI, and further processed using the DADA2 pipeline (version 1.10.1) (Callahan et al., 2016), implemented in R (version 3.5.1). DADA2 was used to trim raw sequences to remove low quality bases and filtered using the following parameters: truncLen = c (290,210), maxEE = c (2,2), trimLeft = c (8,8), minFoldParentOverAbundance = 4 and allowoneoff = TRUE. The filtering was followed by merging the paired-ends and the removal of chimeras from the dataset to create an amplicon sequence variant (ASV) table (Table S1). ASVs were then taxonomically assigned against the SILVA database, release 132 (Quast et al., 2012), using the DECIPHER package, version 2.10.2 (Wright, 2016).

### 2.7 Statistical analyses

Differences in abundances of meiofauna and cable bacteria at the end of the experiment were tested with One-Way ANOVA. Differences in experimental parameters (OPD, H_2_S horizon, O_2_ and H_2_S fluxes) at different times were tested using Two-Way Repeated Measures ANOVA after running normality (Shapiro-Wilk) and equal variance (Brown-Forsythe) tests. When the ANOVA tests showed significant differences (p < 0.05), pairwise post-hoc comparisons among treatments were performed using the Student-Newman-Keuls Method. Statistical analyses of experimental data were performed in SigmaPlot 14.0 (Systat Software, USA). If not stated otherwise in the text, measurements are reported in the results as average ± st.err.

Differences in microbial community composition between treatments were examined by non-metric multidimensional scaling (NMDS) ordination. NMDS ordination was performed using Bray-Curtis dissimilarity matrix based on relative abundances of microbial ASVs and plotted with the “plot_ordination” function of the phyloseq R package (McMurdie and Holmes, 2013), respectively. Statistically significant effects of meiofauna abundance on microbial community composition were examined using a permutational multivariate analysis of variance (PERMANOVA) with the *adonis* function of the vegan package (Oksanen et al., 2018). Differences in community composition of sulfur oxidizing bacteria (SOB) due to meiofauna abundances was examined using the same procedure described above after sub-setting the data for relevant taxa (Table S2) (Wasmund et al., 2017).

## 3 Results

### 3.1 Meiofauna and cable bacteria abundances

**Fig. 1:**
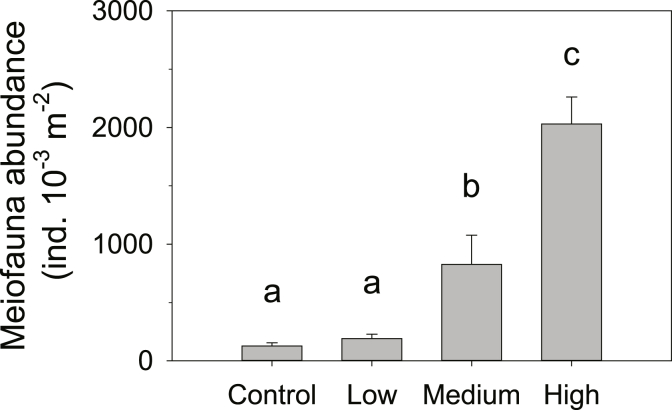
Meiofauna abundances in the four treatments. Different letters indicate significant differences (Kruskal-Wallis ANOVA and Student-Newman-Keuls post-hoc test; *p* < 0.05) among treatments. Vertical columns represent average abundances, while error bars represent st.err (*n* = 3 per treatment).

Meiofauna counting at the end of the incubation confirmed that the aimed gradient in meiofauna abundances was successfully established (Fig. 1). The control treatment had the lowest abundance of 128 ± 26 ind. 10^−3^ m^−2^, followed by the low meiofauna treatment 191 ± 38 ind. 10^−3^ m^−2^, the medium meiofauna treatment 828 ± 249 ind. 10^−3^ m^−2^ and the high meiofauna treatment 2030 ± 232 ind. 10^−3^ m^−2^ (Fig. 1). Since we did not count meiofauna abundance also in the extracts, we cannot say whether there was net mortality of meiofauna during the experiments. The meiofauna organisms in both control and manipulated treatments consisted mainly of nematodes (range 78–1793 ind. 10^−3^ m^−2^) and kinorynchs (range 0–146 ind. 10^−3^ m^−2^) (Table 1). The other meiofaunal groups—cladocerans (mainly bosminidae), copepods (mainly harpacticoids), halacaroids, and ostracods—were found at substantially lower abundances (Table 1). Meiofauna abundances were significantly different between treatments (ANOVA, *p* = 0.022) (Table 2). Abundances were significantly higher in the high meiofauna compared to medium, low and control (Fig. 1), they were significantly higher in the medium meiofauna compared to low and control (Fig. 1), but were not significantly different between low treatment and control (Fig. 1).

**Table 1:** Average abundances of meiofauna organisms and filaments of cable bacteria in top 0–1 cm sediment layer of the four experimental treatments (±st.err; n = 3). Meiofauna abundance values are in ind. 10^−3^ m^−2^. Cable bacteria filament abundances are in m cm^−2^.

Organism	Control		Low		Medium		High	
	avg	st.err	avg	st.err	avg	st.err	avg	st.err
Cladocera	33	6	27	2	90	42	57	16
Copepoda	16	5	17	3	16	4	21	8
Halacaridae	0	0	4	2	3	3	4	2
Kinorhyncha	0	0	0	0	40	15	146	31
Nematoda	78	18	142	35	662	196	1793	229
Ostracoda	0	0	0	0	16	13	9	2
*Total meiofauna*	*128*	*26*	*191*	*38*	*827*	*249*	*2030*	*232*
*Cable bacteria filaments*	*119*	*12*	*112*	*7*	*96*	*9*	*113*	*10*

**Table 2:** Summary of the results of the One-Way ANOVA (meiofauna, cable bacteria) and the Two-Way Repeated Measures ANOVA performed to test the effect of factors Time and Treatment on dependent variables (OPD = oxygen penetration depth, sulfidic horizon, oxygen flux, and sulfide flux). H value (meiofauna) depicts results from the Kruskal-Wallis One-Way ANOVA.

Variable	Factor	Df	Test result	*p*
Meiofauna abundance	–	3	H = 9.667	0.022
Cable bacteria abundance	–	3	F = 0.963	0.456
OPD	Time	2	F = 4.286	0.026
	Treatment	3	F = 23.789	<0.001
	Time x Treatment	6	F = 6.838	<0.001
Sulfidic horizon	Time	2	F = 1.333	0.282
	Treatment	3	F = 28.151	<0.001
	Time x Treatment	6	F = 6.108	<0.001
Oxygen flux	Time	2	F = 0.125	0.886
	Treatment	3	F = 17.312	0.002
	Time x Treatment	6	F = 1.907	0.161
Sulfide flux	Time	2	F = 21.059	<0.001
	Treatment	3	F = 14.277	<0.001
	Time x Treatment	6	F = 6.974	<0.001

Cable bacteria were detected in all sediments by means of FISH analyses (Table 1). There were no significant differences in cable bacteria filament abundances among treatments (*p* = 0.456) (Table 2). Similar abundances of cable bacteria between treatments ensured that the effects between treatments were due to the meiofauna gradient.

### 3.2 Interpretation of solute profiles

**Fig. 2:**
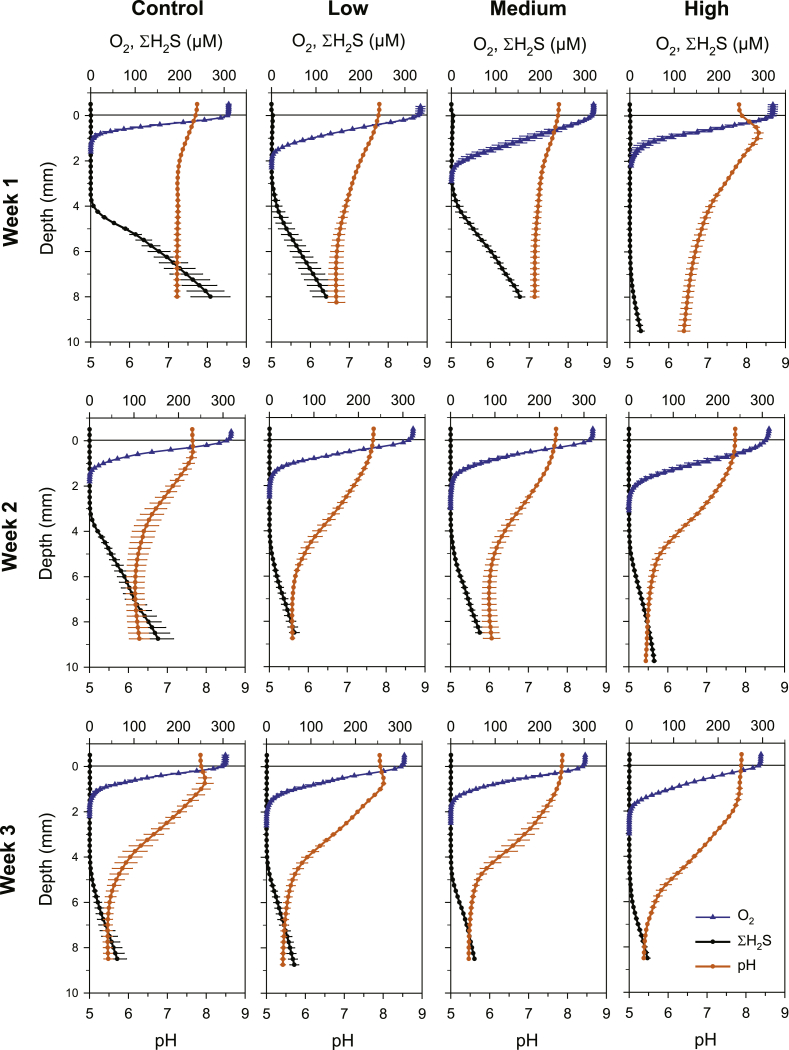
Sediment concentration microprofiles of O_2_ (blue), ΣH_2_S (black) and pH (orange) measured in the four treatments at three different time points. Values are given as average ± st.err (each profile is *n* = 9). (For interpretation of the references to colour in this figure legend, the reader is referred to the Web version of this article.)

**Fig. 3:**
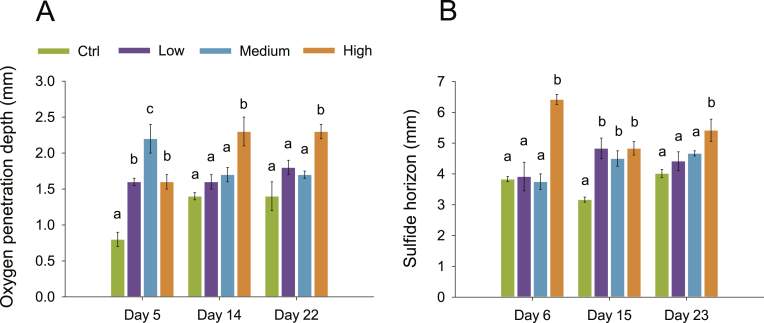
A) Oxygen penetration depths (OPDs) and B) Depth of sulfide horizons measured with microsensors in the four treatments. Different letters on top of each bar indicate significant differences (Two-Way Repeated Measures ANOVA and Student-Newman-Keuls post-hoc test; *p* < 0.05) among treatments. Bars represent average values ± st.err (each bar is *n* = 9).

Sediments had porosity ranging 0.85–0.93 and organic matter content ranging 15–19%. An overview of all average solute profiles recorded for this study is given in Fig. 2. During the course of the experiment O_2_ concentration in the overlying water never dropped below in situ value of 280 μM, and it generally ranged between 300 and 330 μM (Fig. 2). After 5 days of incubation, there were statistically significant differences in oxygen penetration depth (OPD) between treatments (*p* < 0.001) (Table 2). OPD was 0.8 ± 0.1, 1.6 ± 0.1, 2.2 ± 0.2 and 1.6 ± 0.1 mm in the control, low, medium and high meiofauna treatments, respectively (Fig. 3A). Low, medium and high meiofauna treatments had significantly (*p* < 0.001) higher OPDs compared to control (Fig. 3A). After 14 days of incubation, OPDs were 1.4 ± 0.1, 1.6 ± 0.1, 1.7 ± 0.1 and 2.3 ± 0.2 mm in control, low, medium and high meiofauna treatments, respectively (Fig. 3A). The high meiofauna treatment significantly deepened oxygen penetration compared to control (*p* < 0.001), low (*p* = 0.002) and medium (*p* = 0.004) meiofauna treatments (Fig. 3A). After 22 days of incubation, OPD values displayed the same pattern as that at day 14 and were 1.4 ± 0.2, 1.8 ± 0.1, 1.7 ± 0.1 and 2.3 ± 0.1 mm in control, low, medium and high meiofauna treatments, respectively (Fig. 3A). Again, the high meiofauna treatment had significantly deeper oxygen penetration than control (*p* < 0.001), low (*p* = 0.008) and medium (*p* = 0.007) meiofauna treatments (Fig. 3A).

The pH ranged between 7.6 and 7.9 at the sediment-water interface and decreased with depth in all treatments (Fig. 2). At day 6, pH stabilized between 6.7 and 7.2 at 6 mm depth in the control, low, medium meiofauna treatments. In the high meiofauna treatment there was a pH maximum of 8.3 at 0.75 mm depth and it decreased to 6.4 at 9.5 mm depth (Fig. 2). At day 15, sediments became more acidic with pH values decreasing to 5.6–6.2 at 8 mm depth in the control, low, medium meiofauna treatments. Again, the high meiofauna sediment had the lowest pH values of 5.4 at 9 mm depth. An almost imperceptible pH maximum (pH 7.7) was forming in the control sediment (Fig. 2). At day 23, all pH profiles decreased to 5.4 at around 8 mm depth. Subsurface pH maxima (pH 8.0) were recorded in the control and low meiofauna treatments (Fig. 2).

Depth of the sulfide horizon, which defines the total volume of oxidized sediment, was significantly different between treatments (*p* < 0.001) (Table 2). The depth trends of the sulfide horizons were generally following that of OPDs (cf., Fig. 3A and 3B). After 6 days of incubation, sulfide appeared at 3.8 ± 0.1, 3.9 ± 0.5, 3.8 ± 0.3 and 6.4 ± 0.2 mm in control, low, medium and high meiofauna, respectively (Fig. 3B). High meiofauna increased significantly the H_2_S horizon depth comparatively to the other treatments (*p* < 0.001) (Fig. 3B). High meiofauna coincided with a 68% increase in the volume of oxidized sediment compared to control sediment. At day 15, the sulfide horizons were 3.2 ± 0.1, 4.8 ± 0.3, 4.5 ± 0.3, 4.8 ± 0.2 mm in control, low, medium and high meiofauna, respectively (Fig. 3B). The three meiofauna treatments had significantly deeper sulfide horizons compared to control (*p* < 0.01) (Fig. 3B). At day 23, the sulfide horizons increased both in high meiofauna and in control, while in low and medium meiofauna they remained relatively constant. The horizons were 4.0 ± 0.1, 4.4 ± 0.3, 4.7 ± 0.1, 5.4 ± 0.4 mm in control, low, medium and high meiofauna, respectively (Fig. 3B). There was a significant increase in H_2_S horizon in high meiofauna than in the other treatments (*p* < 0.05) (Fig. 3B).

### 3.3 Fluxes of oxygen and sulfide

**Fig. 4:**
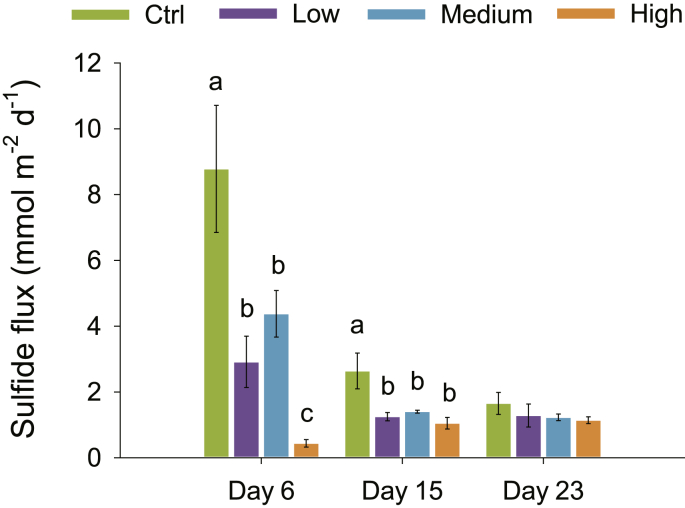
Sulfide fluxes calculated from the ΣH_2_S gradients in the four treatments. Different letters on top of each bar at day 6 and 15 indicate significant differences (Two-Way Repeated Measures ANOVA and Student-Newman-Keuls post-hoc test; *p* < 0.05) among treatments. There were no significant differences at day 23. Bars represent average values ± st.err (each bar is *n* = 9).

Oxygen and sulfide fluxes from the gradients in porewater profiles—calculated with both molecular diffusivity (D_s_) and biodiffusivity (D_b_)—were one or two order of magnitude higher for O_2_ than for H_2_S (cf., Fig. S2 and Fig. 4). Fluxes of O_2_ ranged between −58 and −43 mmol m^−2^ d^−1^ (Fig. S2) and were significantly different between treatments (*p* = 0.002) (Table 2). After 5 days of incubation, fluxes were significantly higher in control compared to the other treatments (Fig. S2). At the other time points, O_2_ fluxes were not significantly different between treatments (Fig. S2). There was no correlation between O_2_ fluxes and meiofauna abundance (data not shown).

Fluxes of ΣH_2_S ranged between 8.8 and 0.4 mmol m^−2^ d^−1^ (Fig. 4) and were significantly different between treatments (*p* < 0.001) (Table 2). The most striking differences were found after 6 days of incubation, when fluxes were 8.8 ± 1.9, 2.9 ± 0.8, 4.4 ± 0.7, 0.4 ± 0.1 mmol m^−2^ d^−1^ in control, low, medium and high meiofauna, respectively (Fig. 4). Fluxes were significantly higher in control compared to the other treatments, while high meiofauna further significantly lowered fluxes compared to low and medium abundances (all with *p* < 0.001) (Fig. 4). After 15 days of incubation, sulfide fluxes were significantly higher in control compared to the other treatments (all with *p* < 0.05), while at the end of the incubation fluxes were not significantly different anymore (Fig. 4)

### 3.4 Microbial diversity and community structure

**Fig. 5:**
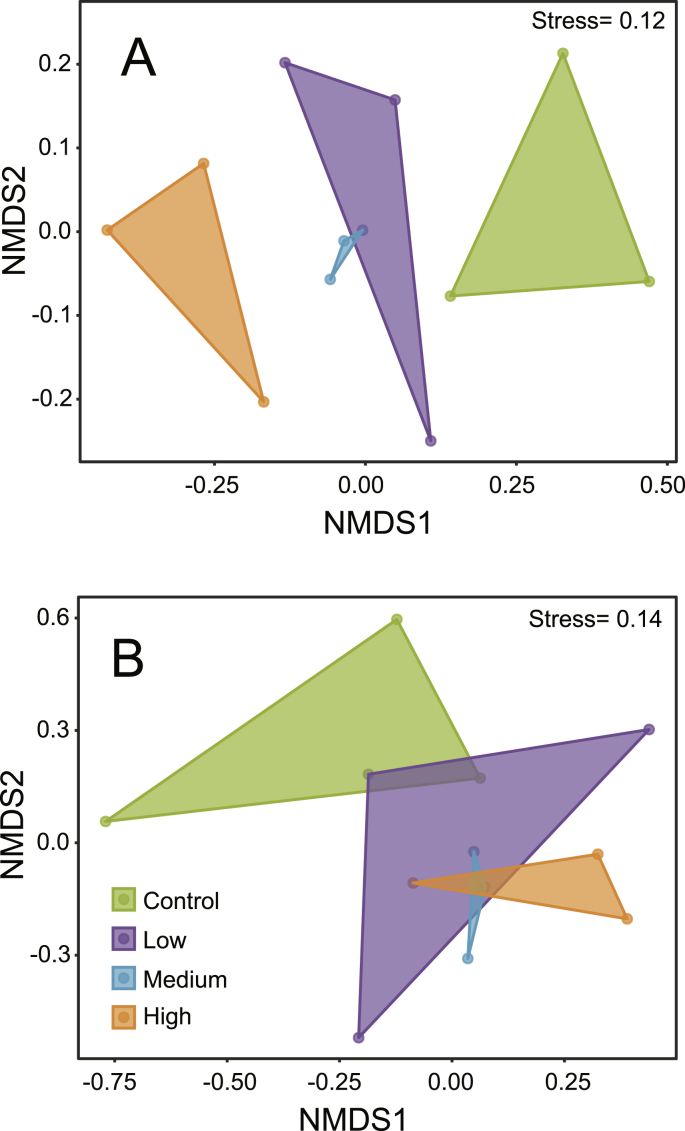
Non-metric multidimensional scaling (NMDS) ordination based on whole microbial community (A) and on sulfide oxidizing bacterial community (B). Control = control treatment; Low = low meiofauna treatment; Medium = medium meiofauna treatment; High = high meiofauna treatment.

Non-metric multidimensional scaling (NMDS) ordination based on relative abundances of the 16S rRNA marker gene showed that samples belonging to different treatments formed distinctive clusters (Fig. 5A). A PERMANOVA analysis (*adonis* pseudo-F_3,11_ = 1.4; p = 0.001) indicated a significant linkage between meiofauna abundances and the microbial community structure. A similar pattern was found for the SOB community (Fig. 5B), where a significant effect of treatment was also detected (*adonis* pseudo-F_3,11_ = 1.2; p = 0.017). In the SOB community, the highest relative abundance was reached by *Pseudomonas* and by the cable bacteria genus *Candidatus* Electrothrix (Fig. S3).

## 4 Discussion

### 4.1 Meiofauna effect on oxygenation and sulfide removal

Overall, our results show that high densities of meiofauna increased the depth of both oxygen penetration and sulfide horizon, and thus the total volume of oxidized, sulfide-free sediment. Similar abundances of cable bacteria filament between treatments at the end of the experiment emphasize that observed effects were caused by differences in meiofauna. However, there were important temporal trends in these effects. On the short-term—5 to 6 days after animals’ colonization—high meiofauna bioturbation increased the volume of sulfide-free sediment by 68%. The higher abundances of meiofauna—which were mainly represented by nematodes—may have different types of effects on sulfide removal. Most importantly, increase in meiofauna densities enhance bioturbation and the OPD. This increase in sediment oxygenation is in line with previous studies, showing that meiofauna activity doubles rates of solute transport in the top oxic sediment layer (Aller and Aller, 1992), and thereby increases OPD (Rysgaard et al., 2000).

Meiofaunal organisms, which principally move through sediment particles interstices (Giere, 2009), increase porosity (Aller and Aller, 1992), enhance sediment mixing and may stimulate iron (Fe) and/or manganese (Mn) cycling, which leads to removal/precipitation of free H_2_S. This effect has been reported for macrofaunal digging and irrigation (e.g., Bonaglia et al., 2019; Seitaj et al., 2015), and our results suggest that the same mechanism may happen due to high bioturbation by meiofauna. However, we cannot exclude that in our sediments nematodes were also oxidizing sulfide thanks to their microbial ecto- and endosymbionts (Giere et al., 1995; Hentschel et al., 1999; Ott et al., 1991; Polz et al., 1992). Bacterivory is the most abundant feeding type among nematodes collected in the same area (Broman et al., 2019; Olafsson and Elmgren, 1997). These nematodes can migrate several times per day between the oxic and sulfidic layers to promote the activity of their symbionts from which they receive food (Ott et al., 1991). Thus, these animals may have played a direct role in the detoxification of the sulfide-rich environment. On the medium- and long-term—14 to 23 days after animals’ colonization—meiofauna continued to exert a control on the extension of the oxic and sulfide-free zone.

### 4.2 Meiofauna and cable bacteria alteration of sulfide oxidation

Besides expanding the extent of the sulfide-free zone, the presence of meiofauna substantially decreased the fluxes of sulfide after six days of incubation. However, the rapid decline of the fluxes in the control during the course of the experiment indicated the onset of an additional mechanism of sulfide oxidation. By performing electrogenic sulfur oxidation (e-SOx) cable bacteria can couple sulfide oxidation to distant oxygen reduction thereby generating a separation zone between the oxic and sulfidic zones in the sediment (Nielsen et al., 2010), and a decline of the upwards fluxes of free sulfide over time (Schauer et al., 2014). FISH analysis revealed that cable bacteria were present at comparable densities in all our treatments and control at the end of the experiment. Such densities were in the same range as those recently reported for seasonally hypoxic coastal Baltic sediments (Hermans et al., 2019), and higher than those from almost anoxic or fully oxic Baltic sites (Marzocchi et al., 2018). A diagnostic feature of e-SOx is the pronounced proton consumption in the oxic zone due to the high proton demand of electrochemical O_2_ reduction and acidification at the depth of sulfide consumption, due to the net proton produced by electrochemical sulfide oxidation (Meysman et al., 2015). These trends were visible throughout the incubation period (Fig. 2). FISH and geochemical evidences therefore converge in indicating that the decrease in the upward flux of H_2_S recorded transversally in our treatments and control during the experiment may be attributed to the activity of cable bacteria.

Previous studies indicate that the physical disturbance and alteration of chemical gradients as induced by macrofauna can inhibit cable bacteria activity (Malkin et al., 2014). To date, there are no studies reporting on meiofauna interactions with cable bacteria and it has only been speculated that at high abundances, nematodes could potentially be important in grazing cable bacteria in well-oxygenated muddy sediments (Aller et al., 2019). Our results show that contrarily to macrofauna, meiofauna can coexist with a consistent population of cable bacteria and therefore that sediment reworking and potential predation by meiofauna do not impede cable bacteria activity. Since cable bacteria abundance was similar along the meiofauna gradient, the overall interaction between meiofauna and cable bacteria appears to be rather neutral. It is however noteworthy that after six days of incubation, the high meiofauna treatment showed higher sulfide removal and a significant decrease in sulfide flux compared to the other treatments, and that this was coupled to a more pronounced pH signature of e-SOx (marked pH maxima in the oxic zone). This is suggestive of a possible positive interaction between the cable bacteria and meiofauna, where particle reworking and mobilization of solutes by meiofauna (Aller and Aller, 1992; Bonaglia et al., 2014; Rysgaard et al., 2000) might have increased the net transport of oxygen into the sediment and consequently accelerated the establishment of the cable bacteria population.

The most striking differences in solute fluxes were determined 5–6 days after incubation started, i.e., when visual investigations at the stereomicroscope revealed that meiofauna were most active (data not shown). At this time point, sulfide fluxes were 8.8 and 0.4 mmol S m^−2^ d^−1^, while oxygen fluxes were −58 and −42 mmol O_2_ m^−2^ d^−1^ in control and high meiofauna treatments, respectively. Since sulfide was not escaping to the water column, it was either buried as minerals (iron sulfides and pyrite) or reoxidized to sulfur and sulfate (Berner, 1984). Assuming that the process of sulfide oxidation to sulfate has an O_2_:S stoichiometry of 2:1, the calculated sulfide fluxes would determine O_2_ consumptions of −17.6 and −0.8 mmol O_2_ m^−2^ d^−1^ in control and high meiofauna, respectively. Theoretically, this means that ca. 30% of the O_2_ consumed in the control was actually used to oxidize sulfides, likely by e-SOx. In the high meiofauna treatment, however, only 2% of the O_2_ consumption was used by the sulfide oxidation process. Here sediment acidification as induced by e-SOx might have promoted the dissolution of FeS minerals with the consequent release of H_2_S and Fe^2+^ (Risgaard-Petersen et al., 2012), thereby adding two additional sinks of oxygen. An additional fraction of O_2_ must have been used in the processes of reoxidation of other reduced compounds such as amorphous and mineral ferrous and manganous compounds by microbial oxide reductions, ammonium via nitrification, and eventually methane (CH_4_) through CH_4_ oxidation. Previous studies have suggested that meiofauna and protozoans can increase nitrification activity (Bonaglia et al., 2014; Prast et al., 2007). There are actually no studies quantifying CH_4_ oxidation rates in relation to meiofauna bioturbation. However, if we extend our findings of H_2_S oxidation to other gases, this may suggest that increased bioturbation activity due to high meiofauna abundances has the potential to decrease benthic CH_4_ fluxes.

### 4.3 Effects of meiofauna abundance on microbial diversity

Beta diversity was significantly different among treatments, and the largest differences were clearly between control and high meiofauna clusters (Fig. 5), which strongly suggest that increasing abundances of meiofauna exert a dominant control on microbial communities. Our results showed that high meiofauna abundances increased the bioturbation intensity and solute advection (D_b_), resulting in significant differences in geochemical conditions, which likely resulted in different microbial communities. Lab experiment showed that bacterivorous nematodes increase bacterial densities (Hubas et al., 2010) and introduce diversity in the bacterial community under high grazing pressure (De Mesel et al., 2004), which indicate that high nematode abundances actually stimulate bacterial growth.

It was suggested that meiofauna affect microbial community structure by exerting a top-down control on bacteria (Nascimento et al., 2012; Näslund et al., 2010). Those studies may have disturbed the natural occurring processes and communities by sediment sieving, while our sediments were intact and represent more realistic conditions for the development of microbial bacterial communities. Our results also indicate that meiofauna have a significant top-down influence on the chemolithotrophic SOB communities and that this happened even at low meiofauna densities. Looking at the different genera constituting the SOB community, it was evident that after the ubiquitous *Pseudomonas*, the most abundant SOB in the four treatments was *Candidatus* Electrothrix, the mostly marine genus of cable bacteria (Trojan et al., 2016).

## 5 Conclusions

Meiofaunal organisms are widespread in all sediments, and reach high diversity and abundances even in seasonally hypoxic systems (Elmgren, 1975; Sergeeva et al., 2013; Wetzel et al., 2001). They are fast growing and have short generation times of few weeks, which allow them to increase their abundance in relatively short time (Giere, 2009; Warwick, 1981). Here we show that meiofauna—and their hitherto neglected coexistence with cable bacteria—play an important role in a number of geochemical processes as they: (1) increase the oxygen penetration depth; (2) decrease the overall sulfide flux; and (3) increase the volume of oxidized, sulfide-free sediment in seasonally hypoxic environments. Moreover, we show that high meiofauna abundances and bioturbation structure microbial diversity of hypoxic sediments. These aspects have two pivotal implications. Meiofauna prevent sulfide emission to the water column where it could cause deleterious effects on marine life. Meiofauna and cable bacteria—upon oxygenation—greatly facilitate recolonization by macrobenthos and fish. Our results answer but also raise intriguing questions about potential top-down effects of meiofauna on microbial detoxification of sulfides via chemosynthetic symbionts, free-living SOB and cable bacteria. To address such questions, future studies investigating how meiofauna behavior and functional traits affect benthic biogeochemical processes and metabolic pathways would be very useful, particularly when coupled to geochemical analyses and modern molecular techniques.

## CRediT authorship contribution statement

**Stefano Bonaglia:** Conceptualization, Investigation, Formal analysis, Data curation, Writing - original draft, Writing - review & editing. **Johanna Hedberg:** Investigation, Formal analysis, Writing - original draft. **Ugo Marzocchi:** Conceptualization, Writing - original draft, Writing - review & editing. **Sven Iburg:** Formal analysis, Writing - original draft. **Ronnie N. Glud:** Conceptualization, Writing - original draft. **Francisco J.A. Nascimento:** Conceptualization, Data curation, Writing - original draft, Writing - review & editing.

## Declaration of competing interest

The authors declare that they have no known competing financial interests or personal relationships that could have appeared to influence the work reported in this paper.
