# Supplementary material for: Meiofauna improve oxygenation and accelerate sulfide removal in the seasonally hypoxic seabed
Source: Mar Environ Res. 2020 Jul;159:104968. doi: 10.1016/j.marenvres.2020.104968 (PMC7369627; doi:10.1016/j.marenvres.2020.104968)
Supplement: Multimedia component 1 [file mmc1.docx]

# Meiofauna improve oxygenation and accelerate sulfide removal in the seasonally hypoxic seabed

Stefano Bonaglia, Johanna Hedberg, Ugo Marzocchi, Sven Iburg,

Ronnie N. Glud, Francisco J. A. Nascimento

# Supplementary Information


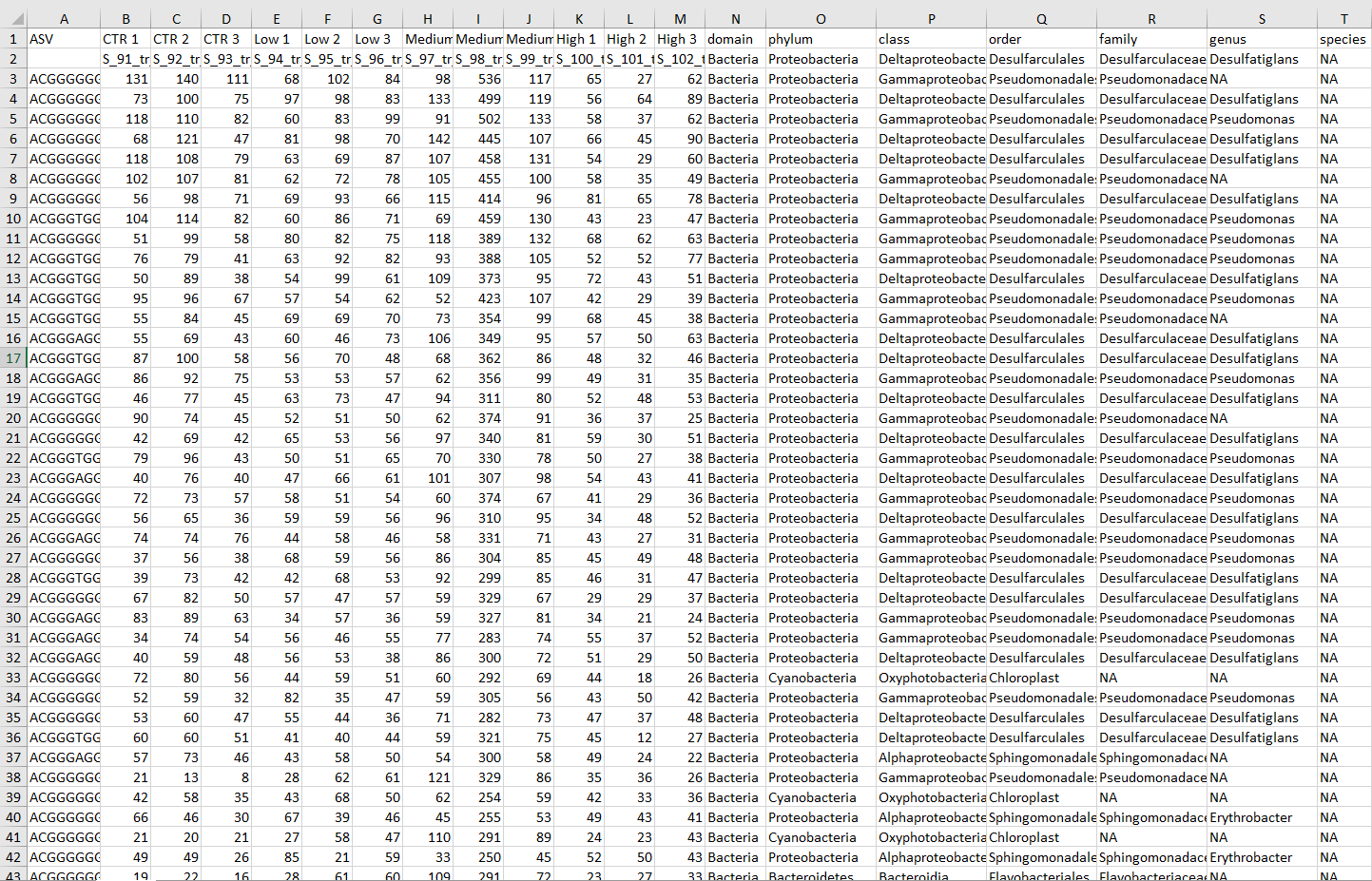


**Table S1** – Layout of supplementary table 1 with all amplicon sequence variants (ASV). For full display of Table 1, refer to the linked Excel file.

**Table S2 –** List of sulfur oxidizing bacteria (SOB) that were considered in the analysis after Wasmund et al. ([2017](#_ENREF_3)).


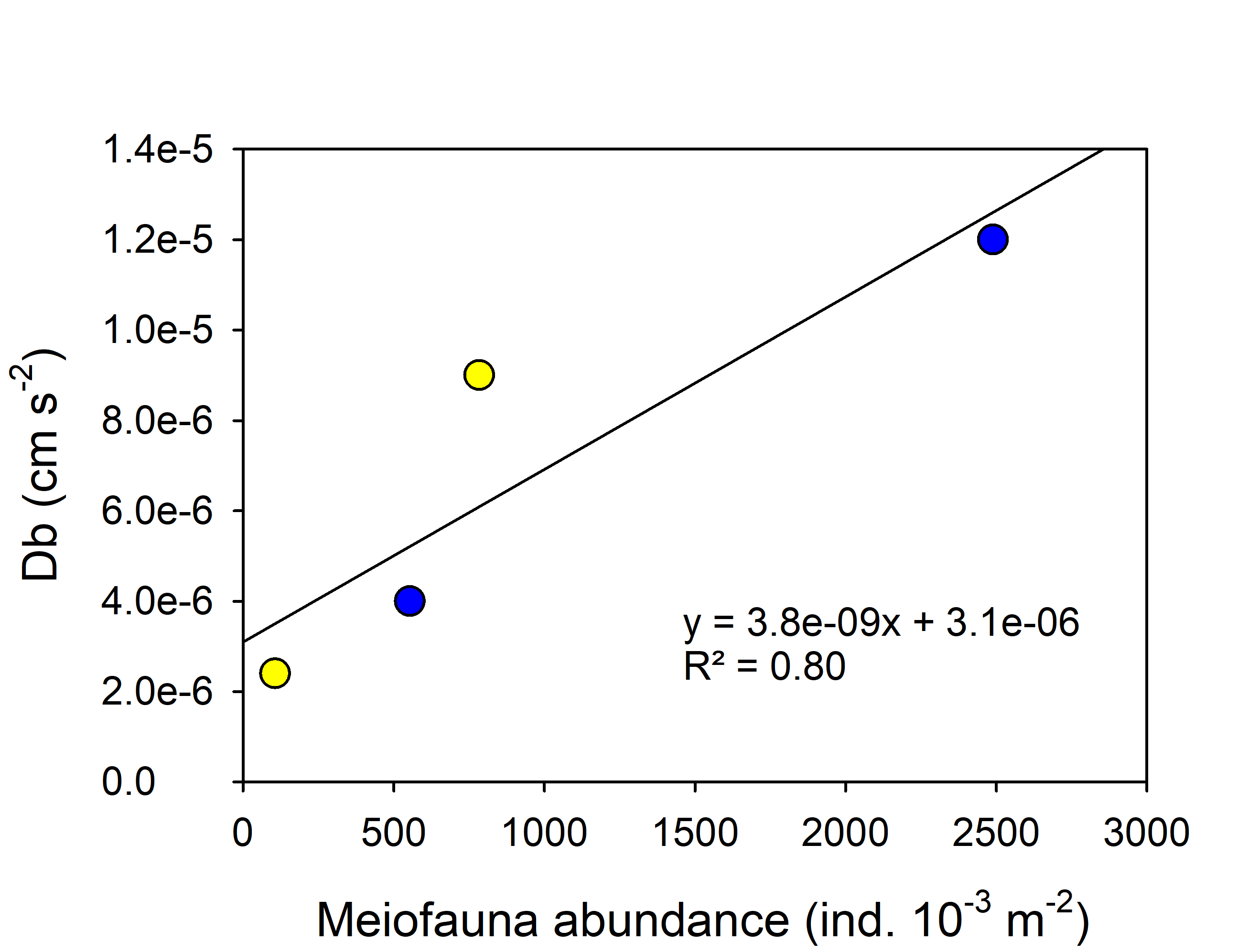


**Figure S1 –** Linear regression between meiofauna abundances and bioturbation coefficients (D_b_) obtained by plotting previously published D_b_ values against actual meiofauna abundances in the experimental sediments. Yellow dots represent values from Bonaglia et al. ([2014](#_ENREF_1)) and blue dots represent values from Rysgaard et al. ([2000](#_ENREF_2)).


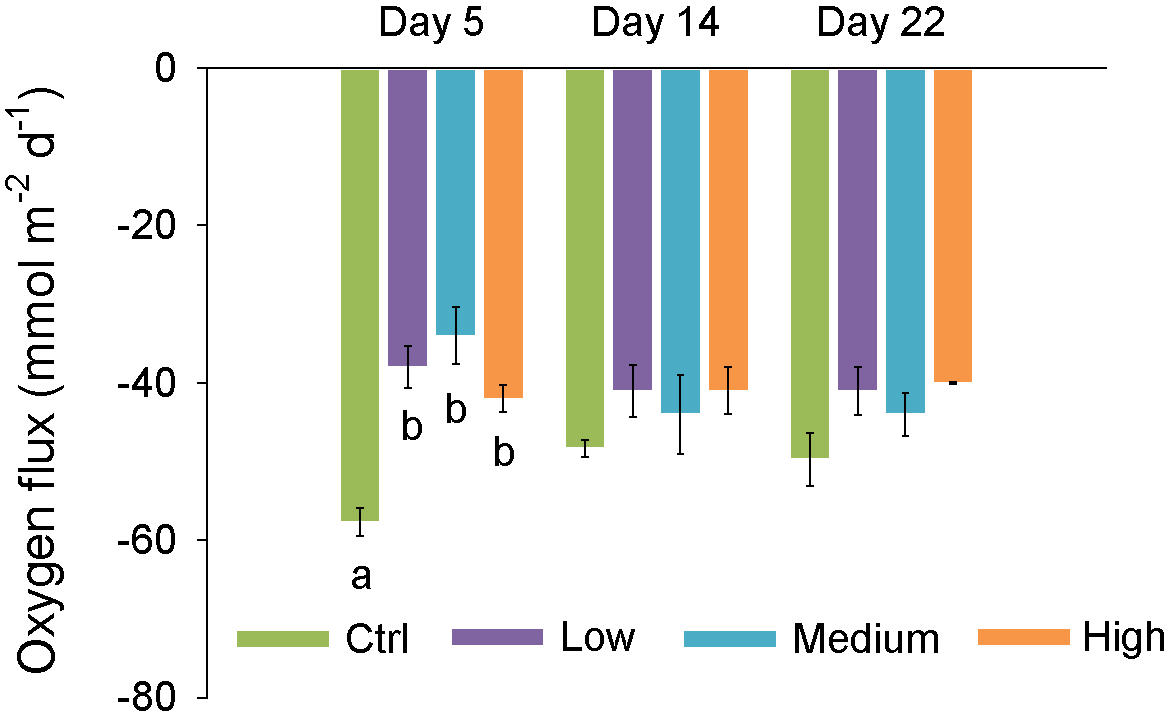


**Figure S2 –** Oxygen fluxes calculated from the O_2_ gradients in the four treatments. Different letters on top of each bar at day 5 indicate significant differences (Two-Way Repeated Measures ANOVA and Student-Newman-Keuls post-hoc test; *p* < 0.05) among treatments. There were no significant differences at day 14 and day 22. Bars represent average values ± st.err (each bar is *n* = 9).


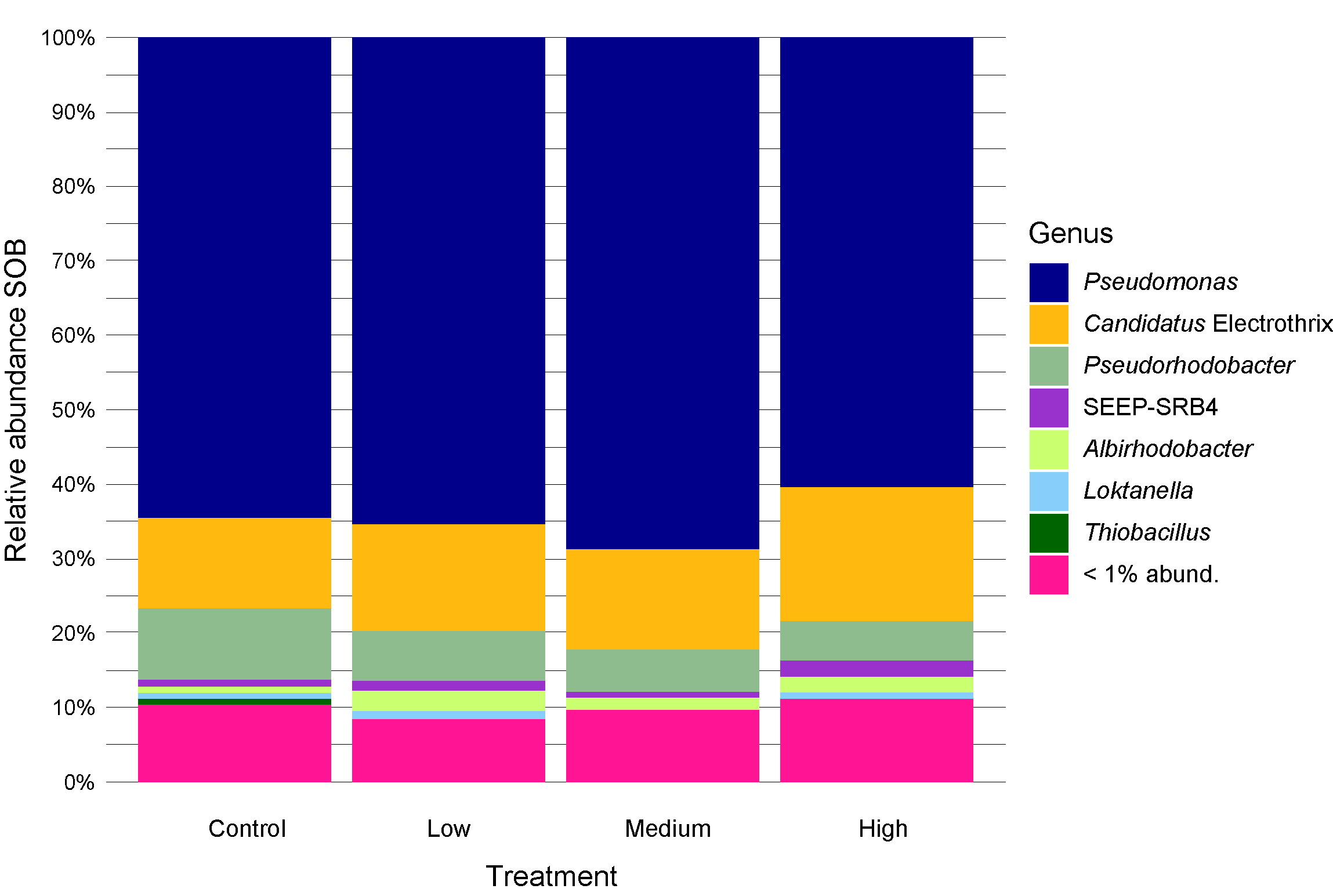


**Figure S3** – Stacked bars of average (*n* = 3) relative abundances (%) of sulfur oxidizing bacteria (SOB) grouped into genera in the four different treatments at the end of the experiment (23 days of incubation). The data was delimited to groups > 1 %, with “< 1 % abund.” showing the remaining genera belonging to groups < 1 %. Labels denote: Control = control; Low = low meiofauna; Medium = medium meiofauna; High = High meiofauna.

## References

Bonaglia, S., Nascimento, F.J.A., Bartoli, M., Klawonn, I., Bruchert, V., 2014. Meiofauna increases bacterial denitrification in marine sediments. Nat. Commun. 5, 5133.

Rysgaard, S., Christensen, P.B., Sorensen, M.V., Funch, P., Berg, P., 2000. Marine meiofauna, carbon and nitrogen mineralization in sandy and soft sediments of Disko Bay, West Greenland. Aquat. Microb. Ecol. 21, 59-71.

Wasmund, K., Mußmann, M., Loy, A., 2017. The life sulfuric: microbial ecology of sulfur cycling in marine sediments. Environ. Microbiol. Rep. 9, 323-344.
